# Coinfection rates and clinical outcome data for cytomegalovirus and Epstein‐Barr virus in post‐transplant patients: A systematic review of the literature

**DOI:** 10.1111/tid.13396

**Published:** 2020-07-27

**Authors:** Colin Anderson‐Smits, Erin R. Baker, Ishan Hirji

**Affiliations:** ^1^ Shire, A Takeda Company Cambridge MA USA; ^2^ CTI Clinical Trial & Consulting Services Covington KY USA

**Keywords:** cytomegalovirus, Epstein‐Barr virus, hematopoietic stem cell transplant, solid organ transplant

## Abstract

**Background:**

In transplant recipients, cytomegalovirus (CMV) infection increases morbidity and mortality; furthermore, coinfection with other human herpesviruses like the Epstein‐Barr virus (EBV) may complicate their management. This systematic literature review aimed to summarize rates of CMV‐EBV coinfection and associated clinical outcomes among solid organ transplant (SOT) and hematopoietic stem cell transplant (HSCT) recipients.

**Methods:**

An electronic literature search was performed using pre‐specified search strategies (January 1, 2010‐October 31, 2018) and following established/best practice methodology. Of 316 publications identified, 294 did not report CMV‐EBV coinfection and were excluded. Studies meeting the inclusion criteria were further analyzed. Due to limited reporting/heterogeneity, data were not meta‐analyzable.

**Results:**

Nine studies (six SOT; three HSCT) reported CMV‐EBV coinfection; rates of coinfection post transplantation varied between 2.6% and 32.7%. Two studies indicated CMV reactivation to be an independent variable associated with EBV reactivation. Among SOT studies, higher rates of graft dysfunction (47.4% vs 22.9%), rejection episodes (20.0% vs 8.9%), or acute rejection (50.0% vs 31.0%) were reported for patients with coinfection than without. In HSCT studies, patients with graft‐vs‐host disease were not reported separately for coinfection. Two studies described cases of post‐transplant lymphoproliferative disorder (PTLD) in patients with CMV‐EBV coinfection and reported rates of PTLD of 92% and 100%.

**Conclusion:**

The CMV‐EBV coinfection rate in HSCT and SOT recipients varied and was associated with increased graft rejection and PTLD compared with patients without coinfection. Further research may improve understanding of the burden of CMV‐EBV coinfection among transplant recipients.

## 1 INTRODUCTION

Ubiquitous in the general population, infection with cytomegalovirus (CMV) is a serious complication of transplantation that increases the risk for allograft rejection, morbidity, and mortality.^1^ The frequency of symptomatic CMV infection among transplant recipients ranges from approximately 8%‐41% in the literature, depending on the allograft received,^1^ serostatus of the donor and recipient, and the use and type of antiviral prophylaxis.^2^ CMV exerts direct cytopathic effects on various organ systems, causing conditions such as pneumonia, gastrointestinal tract disease, and hepatitis.^3^, ^4^ Like other human herpesviruses (HHV), CMV is highly cell‐associated, and transmission requires either intimate mucosal contact or physical transfer of a latent virus via an allograft or leukocyte‐containing blood product.^4^, ^5^ While cytotoxic T lymphocytes form the key host defense system against CMV, failure to reconstitute CMV‐specific cellular immunity following alteration of cell‐mediated immunity heightens the risk of acute CMV disease.^1^ CMV infection can also lead to indirect immunosuppressive effects—including aberrations in T‐cell synthesis, the expression of major histocompatibility antigens, and cytokine and chemokine activity—further increasing the risk of opportunistic infections.^4^, ^6^

CMV is also an active inducer of other HHV.^1^ Through interactions with the transplant recipient's defense system, CMV can enhance the pathogenicity of other viruses, resulting in coinfection and increased cumulative immunosuppression. The interaction between CMV and Epstein‐Barr virus (EBV) has garnered particular attention, given the high seroprevalence of these HHVs in the general population and the tumorigenic role of EBV in the development of post‐transplant lymphoproliferative disorders (PTLDs). Potentially life‐threatening, PTLD encompasses a spectrum of conditions ranging from asymptomatic viremia to true malignancies.^7^ Like CMV, EBV is associated with graft dysfunction and premature graft loss. EBV reactivation is associated with chronic immunosuppression and is likely underestimated in the transplant population.^8^ Although the mechanisms are yet to be fully elucidated, direct virus‐virus interactions may influence disease progression and modulate infectivity by altering gene expression in the cohabitating viruses.^6^

With the growing understanding of virus interactions, the emergence of new viruses, and differing efficacies of current antivirals for HHVs, it is vital to determine the clinical relevance of CMV coinfection with other HHVs, especially coinfection with EBV, following transplantation to devise effective preventative and surveillance strategies. To better understand the clinical burden of CMV and EBV (CMV‐EBV) coinfection in transplant recipients, we performed a systematic literature review (SLR) of the published literature reporting CMV‐EBV coinfection in post‐transplant patients. This study aimed to examine the published literature on the rates of CMV‐EBV coinfection in solid organ transplant (SOT) and hematopoietic stem cell transplant (HSCT) recipients, and to describe the associated clinical outcomes.

## 2 MATERIALS AND METHODS

This study was defined a priori, including the protocol, screening forms, and data extraction templates. The study was performed adhering to the best methods established in the peer‐reviewed science of systematic review research,^9^ and the search and study selection sections of the Preferred Reporting Items for Systematic Reviews and Meta‐Analyses (PRISMA).^10^, ^11^, ^12^

### 2.1 Literature search

An electronic search was performed in MEDLINE^®^ via the PubMed interface and EMBASE^®^ for published articles for the period of January 1, 2010, through October 31, 2018. Search strategies were customized for each database (Table 1); filters were applied to retrieve studies conducted in human subjects and published in English. Records retrieved via searches for general studies on post‐transplant viral coinfection and for studies specific to EBV‐CMV coinfection were screened concomitantly. Additionally, bibliographies of articles retrieved for full‐text review and of pertinent review articles were manually searched for other eligible publications. Furthermore, a search in PubMed, as well as manual Internet searches, without restrictions on language or publication type, were performed (May 2018 through October 2018) to capture more recent studies (publisher‐supplied citations) not yet indexed in MEDLINE^®^.

**Table 1:** MEDLINE^®^ search terms and search strategy employed

General search	CMV‐ and EBV‐targeted search
*Steps*: 1) transplant (title/abstract) 2) cytomegalovirus OR CMV OR “herpesvirus 5” 3) coinfect OR coinfected OR coinfection OR co‐infect OR co‐infected OR co‐infection OR co‐occurrence OR reactivation OR reactivate 4) 1 AND 2 AND 3 4 NOT ([news] OR editorial)	*Steps*: 1) transplant (title/abstract) 2) cytomegalovirus OR CMV OR “herpesvirus 5” 3) Epstein–Barr virus OR EBV OR “herpesvirus 4” 4) 2 AND 3 5) coinfect OR coinfected OR coinfection OR co‐infect OR co‐infected OR co‐infection OR co‐occurrence OR reactivation OR reactivate 6) 1 AND 4 AND 5 6 NOT ([news] OR editorial)

Note

Filters were applied to retrieve studies conducted in human subjects and published in English from January 1, 2010, to October 31, 2018. Additional PubMed and manual Internet searches were performed from May 2018 to October 2018 without restrictions on language or publication type.

Abbreviations: CMV, cytomegalovirus; EBV, Epstein‐Barr virus.

Prospective or retrospective studies reporting either of the following outcomes for patients undergoing SOT or HSCT were selected: (a) rate of CMV‐EBV coinfection or (b) clinical outcomes of patients with CMV‐EBV coinfection following transplantation. Studies documenting coinfections defined as symptomatic clinical infections as well as those citing cases of asymptomatic viremia were considered. White papers, editorials/commentaries, reviews or models without presentation of primary data, news articles, and editorials were excluded.

### 2.2 Study screening and data extraction

All references were screened using a two‐level process. First, titles and abstracts were screened (ERB) and then the full text of studies meeting the preliminary criteria (reports, coinfection rates, and/or clinical outcomes associated with CMV‐EBV coinfection). A second author (CA‐S) screened a 10% random sample of excluded articles. Pre‐specified study‐level data were extracted and summarized (Appendix S1).

## 3 RESULTS

### 3.1 Studies

**Figure 1:**
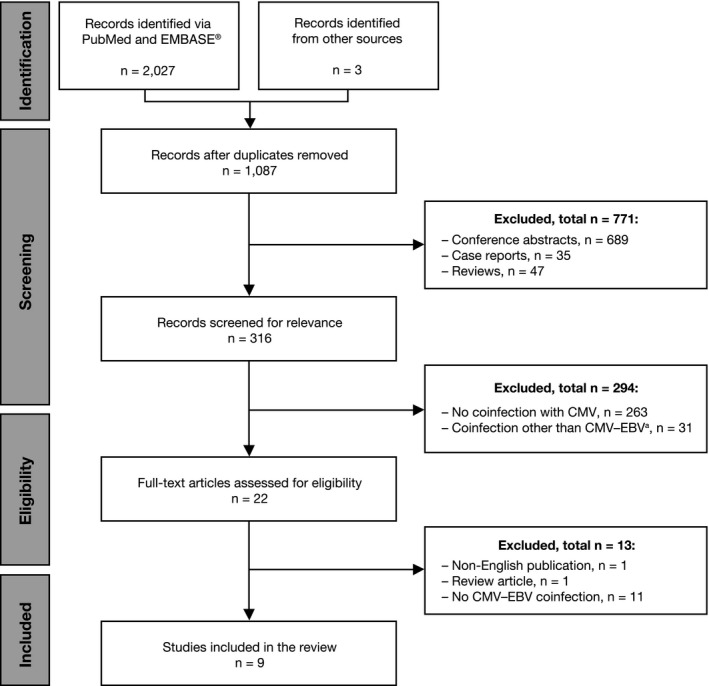
Disposition and selection of publications by screening level and the final number of studies reviewed. ^a^Number of studies reporting coinfections other than CMV‐EBV: pneumonia (3), HIV (4), RSV (1), BK virus (4), *Clostridium difficile* (1), norovirus (1), bacterial enterocolitis (1), hepatitis C (1), HHV (3), HHV‐6 (9), HHV‐7 (3), fungal or mold infections (5), and Merkel cell polyomavirus (1). Some publications reported ≥ 1 agent. These publications included in vivo and in vitro studies. CMV, cytomegalovirus; EBV, Epstein‐Barr virus; HHV, human herpesvirus; HIV, human immunodeficiency virus; RSV, respiratory syncytial virus

The PubMed and EMBASE^®^ searches generated 2027 records. Three additional studies were included from other sources, such as manual searches. After the exclusion of duplicates, conference abstracts, case reports, and reviews, 316 articles were screened for relevance (Figure 1). Of those records, 22 (7%) publications were reviewed in full, and 9 of the 22 (41%) studies were deemed eligible for inclusion.

Table 2 presents an overview of the nine studies identified; six studies assessed SOT recipients (one liver and five renal transplantations),^8^, ^13^, ^14^, ^15^, ^16^, ^17^ and three studies assessed HSCT recipients.^18^, ^19^, ^20^ Two studies estimated infection rates based on retrospective reviews of clinical data.^13^, ^20^ All other studies monitored patients prospectively.

**Table 2:** General characteristics of the studies included in the systematic literature review of post‐transplantation CMV‐EBV coinfection (N = 9)

Study	Study description	Subjects
SOT studies		
*Citation:* Indolfi G, et al 2012^a^ ^,^ ^13^ *Country/region:* King's College Hospital, London, UK (single center)	Review of clinical notes of consecutive cases receiving liver transplants; case series; Apr 2007‐Feb 2008 *Objective:* To investigate the prevalence and timing of EBV and CMV infections during the first 21 d post transplantation in relation to graft function and acute cellular rejection in a large cohort of pediatric liver transplantation recipients treated in a single center	*Sample size:* 62 *Inclusion criteria:* NR *Demographics:* ‐ Mean age: 56.7 (SD: 61.4) mo ‐ Male: 53.2% (33/62)
*Citation:* Bamoulid J, et al 2013^8^ *Country/region:* France (single center)	Patients undergoing renal transplantation; Jan 2002‐Dec 2010 *Objective:* To analyze the natural course of EBV infection in adult kidney transplant recipients, to define risk factors, and to assess the clinical consequences of EBV infection	*Sample size:* 383 *Inclusion criteria:* NR *Demographics:* ‐ Mean age (SD) ▪ No EBV viremia (n = 228): 48 (14.0) y ▪ EBV viremia (n = 155): 49 (14.0) y
*Citation:* Bassil N, et al 2014^15^ *Country/region:* France (single center)	Sub‐analysis of post‐renal transplant patients participating in the phase 3 BENEFIT and BENEFIT‐EXT RCT (belatacept vs CSA); Dec 2005‐May 2007 *Objective:* To compare the incidence of CMV, EBV, BKV, and JC virus (JCV) infections in de novo renal transplant patients receiving either belatacept‐ or CSA‐based immunosuppression	*Sample size:* 62 *Inclusion criteria:* ‐ Patients with ≥ 3 y of follow‐up *Demographics:* ‐ Mean age (SD): ▪ Belatacept (n = 42): 48.4 (13.4) y ▪ CSA (n = 20): 47.5 (15.0) y
*Citation:* Shivanesan P, et al 2016^17^ *Country/region:* India (single center)	Longitudinal, observational cohort study of patients undergoing renal transplantation at a single center (duration of follow‐up: 6 mo) *Objective:* To assess the utility of qRT‐PCR as a diagnostic and monitoring tool for viral infections in post‐renal transplant patients	*Sample size:* 50 *Inclusion criteria:* NR *Demographics:* ‐ Mean (SD) age: 35.1 (10.9) y ‐ Male: 86% (43/50)
*Citation:* Barani R, et al 2018^14^ *Country/region:* India (single center)	Patients who received renal transplants (1997‐2016) and were followed at a nephrology tertiary care center (2011‐2016) *Objective:* To detect EBV by real‑time qPCR in post‐renal transplant recipients and to analyze its co‐occurrence with CMV	*Sample size:* 89 *Inclusion criteria:* ‐ Patients with symptoms suggestive of end‐organ disease, graft dysfunction, or mild elevation of renal parameters without symptoms ‐ Excluded: SCID or asymptomatic with normal renal parameters *Demographics:* ‐ Mean age ± SD (range): 39.2 ± 13.5 (11‐64) y
*Citation:* Blazquez‐Navarro A, et al 2018^16^ *Country/region:* Germany (multicenter)	Sub‐analysis of patients undergoing renal transplantation as part of an RCT (basiliximab vs rabbit ATG; Harmony); Aug 2008‐Nov 2012 *Objective:* To assess the impact and relevance of BKV, CMV, and EBV reactivations during the first year post‐renal transplantation	*Sample size:* 540 *Inclusion criteria:* NR *Demographics:* ‐ Median (IQR) age: 56 (45‐64) y ‐ Male: 64.1% (346/540) ‐ Median (IQR) BMI: 25.8 (23.2‐29.0) kg/m^2^
HSCT studies		
*Citation:* Zallio F, et al 2013^a^ ^,^ ^20^ *Country/region:* Italy (single center)	Patients with advanced hematologic malignancies and allogeneic HSCT; Mar 2005‐Dec 2011 *Objective:* To establish the role of molecular monitoring and preemptive treatment with rituximab post‐HSCT; to investigate the potential association between CMV reactivation and the development of EBV reactivation with respect to including CMV as a risk factor for PTLD	*Sample size:* 101 *Inclusion criteria:* NR *Demographics:* ‐ Median (range) age: 50 (20‐70) y ‐ Male: 54.5% (55/101)
*Citation*: Garcia‐Cadenas I, et al 2015^19^ *Country/region:* Barcelona, Spain (Hospitals Sant Pau and Vall d’Hebrón in Barcelona)	Adult patients undergoing allo‐SCT; Sep 2006‐May 2013 *Objective:* To compare the incidence and prognosis of EBV‐related complications between patients with baseline high‐risk characteristics for PTLD and those with refractory GVHD, prospectively monitored for EBV DNAemia with early rituximab as preemptive therapy	*Sample size:* 133 *Inclusion criteria:* ‐ At high risk for EBV complications, or ‐ Was not at high risk for EBV disease at baseline but developed moderate‐to‐severe acute SR‐GVHD *Demographics:* ‐ Median (range) age: ▪ High risk for EBV (n = 93): 41 (18‐67) y ▪ SR‐GVHD (n = 40): 53 (21‐68) y ‐ Male, %: ▪ High risk for EBV (n = 93): 63.4% (59/93) ▪ SR‐GVHD (n = 40): 62.5% (25/40)
*Citation:* Fan J, et al 2016^18^ *Country/region:* China (single center)	Follow‐up study of patients who received HSCT; Jan‐Jun 2012 *Objective:* To assess the relationships between CMV, EBV, and HHV‐6 infections after HSCT and to identify potential risk factors for viral infection	*Sample size:* 44 *Inclusion criteria:* ‐ IgG‐positive/IgM‐negative CMV *Demographics:* ‐ Median (range) age: 26 (16‐55) y ‐ Male: 52.3% (23/44)

Abbreviations: ATG, anti‐thymocyte globulin; BKV, BK virus; BMI, body mass index; CMV, cytomegalovirus; CSA, cyclosporine A; d, days; EBV, Epstein‐Barr virus; GVHD, graft‐vs‐host disease; HHV‐6, human herpesvirus 6; HSCT, hematopoietic stem cell transplantation; Ig, immunoglobulin; IQR, interquartile range; mo, month; NR, not reported; PTLD, post‐transplant lymphoproliferative disease; qPCR, quantitative polymerase chain reaction; qRT‐PCR, quantitative reverse transcription‐polymerase chain reaction; RCT, randomized controlled trial; SCID, severe combined immunodeficiency disease; SCT, stem cell transplantation; SD, standard deviation; SOT, solid organ transplant; SR‐GVHD, steroid‐refractory graft‐vs‐host disease; y, years.

^a^ Retrospective analysis.

Study samples primarily comprised adults (mean or median age >18 years) with one study in pediatric liver transplantation.^13^ Males represented the majority (52.3%–86%) of patients across all study cohorts. Two studies of renal transplant and HSCT recipients, respectively, selected patients at high risk for EBV disease.^14^, ^19^ Another study included only HSCT recipients who had prior CMV exposure (immunoglobulin [Ig]G‐positive, IgM‐negative CMV).^18^

Table 3 presents the available study data on patient and treatment characteristics pre‐transplant. Five studies reported the pre‐transplant CMV or EBV (or both) serostatus of recipients and donors.^8^, ^15^, ^17^, ^18^, ^19^ While the majority of recipients and donors in these studies were seropositive for both CMV and EBV, 18 of 26 (69.2%) EBV‐seronegative recipients in one study had received renal allografts from EBV‐seropositive donors.^8^

**Table 3:** Patient CMV‐EBV serostatus and treatment characteristics pre‐transplantation, and reported study follow‐up duration

Study	N	Positive CMV‐EBV serostatus, n (%)	Immunosuppression	Antiviral prophylaxis	Duration of follow‐up
SOT studies					
Indolfi G, et al 2012^13^	62	CMV status: (N = 62) IgG−: 28 (45%) IgG+: 14 (23%) Indeterminate: 20 (32%) EBV status: (N = 62) IgG−: 24 (39%) IgG+: 19 (30%) Indeterminate: 19 (30%)	Maintenance therapy only: tacrolimus	Antibacterial and antifungal prophylaxis for ≥ 5 d post transplant Ganciclovir for CMV D^+^/R^−^	Within 21 d post transplant
Bamoulid J, et al 2013^8^	383	EBV 357 recipients were EBV seropositive pre‐transplant EBV serostatus: D^+^/R^+^, 311 D^−^/R^+^, 46 D^+^/R^−^, 18 D^−^/R^−^, 8	Induction: ATG or anti‐CD25 Maintenance: ATG/anti‐CD25 + MMF, tacrolimus, steroid	For CMV R^+^ or D^+^/R^−^ valganciclovir, SMZ‐TMP for 3 mo post transplant For EBV D^+^/R^−^: IVIG for 6 mo post transplant	12 mo planned
Bassil N, et al 2014^15^	62	CMV (+/−): ‐ Belatacept (n = 42) D, 24/18 R, 25/17 ‐ CSA (n = 20): D, 13/7 R, 10/10 EBV (±): ‐ Belatacept (n = 42): D, 39/3 R, 38/4 ‐ CSA (n = 20): D, 18/2 R, 16/4	Induction: basiliximab + MMF+steroid tapered by 6 mo post transplant Maintenance: belatacept or CSA	SMZ‐TMP for 6 mo post transplant For CMV R^+^ or D^+^/R^−^: valganciclovir for 3 mo post transplant	36 mo planned
Shivanesan P, et al 2016^17^	50	CMV D^+^, 96%; R^+^, 98% D^+^/R^+^: 48 (96) D^+^/R^−^: 1 (2) D^−^/R^−^: 1 (2) EBV NR	Tacrolimus or CSA + MMF/azathioprine; basiliximab/ATG induction for cases with unrelated donors 23 (46%) patients received induction therapy	None used	6 mo planned
Barani R, et al 2018^14^	89	NR	NR	NR	Assessed at enrollment only; infections were characterized based on the time of study assessment post transplant: Immediate, 0‐3 mo Late, >3‐12 mo Very late, >1 year
Blazquez‐Navarro A, et al 2018^16^	540	NR	Induction: basiliximab or rabbit ATG Maintenance: tacrolimus + MMF ±steroids	For CMV or EBV D^+^/R^−^: valganciclovir ≥ 3 mo	12 mo planned
HSCT studies					
Zallio F, et al 2013^20^	101	CMV, n = 100 D^−^/R^−^, 20 (20) D^−^/R^+^, 24 (24) D^+^/R^+^, 33 (33) D^+^/R^−^, 23 (23) EBV NR	Myeloablative conditioning (n = 53) or reduced‐intensity conditioning (n = 48) In vivo T‐cell depletion (n = 46) GVHD prophylaxis with CSA + methotrexate or MMF	Preemptive anti‐CMV therapy in cases with ≥ 1 positive cell/200,000 leukocytes confirmed twice	Median (range): 13 (0‐78) mo
Garcia‐Cadenas I, et al 2015^19^	404	CMV D^−^/R^−^ Baseline cohort: 18/93 (19.4) SR‐GVHD cohort: 3/40 (7.5) EBV NR	Myeloablative conditioning (CY + TBI/BU) or reduced‐intensity conditioning; ATG for cases with mismatched unrelated donors Conditioning regimen for cord‐blood transplants T‐cell depletion during conditioning	Antiviral, antifungal, SMZ‐TMP, or nebulized pentamidine Preemptive rituximab for EBV > 1000 copies/mL on two consecutive occasions or a single detection of EBV > 2,000 copies/mL	Median of ~ 20 mo (median [range] 604 [34‐2204] d)
Fan J, et al 2016^18^	44	CMV IgG + IgM−, 44 (100) EBV NR	±ATG with MMF + CSA with short‐term methotrexate and ganciclovir for 7 d pre‐transplant T‐cell depletion reported	SMZ + ganciclovir	3‐12 mo planned

Note

All data are as reported in the original publications.

Abbreviations: ATG, anti‐thymocyte globulin; BU, busulfan; CD, cluster of differentiation; CMV, cytomegalovirus; CSA, cyclosporine A; CY, cyclophosphamide; D, donor; d, day; EBV, Epstein‐Barr virus; GVHD, graft‐vs‐host disease; HSCT, hematopoietic stem cell transplantation; Ig, immunoglobulin; IVIG, intravenous immunoglobulin; MMF, mycophenolate mofetil; mo, month; NR, not reported; R, recipient;SMZ‐TMP, sulfamethoxazole‐trimethoprim; SOT, solid organ transplant; SR‐GVHD, steroid‐refractory graft‐vs‐host disease; TBI, total body irradiation.

Five studies reported a planned duration of follow‐up^8^, ^15^, ^16^, ^17^, ^18^ (6 months^17^ to 36 months^15^). Two studies reported median follow‐up durations (13 and ~20 months),^19^, ^20^ and two studies reported limited testing.^13^, ^14^

### 3.2 CMV‐EBV coinfection post transplantation

**Figure 2:**
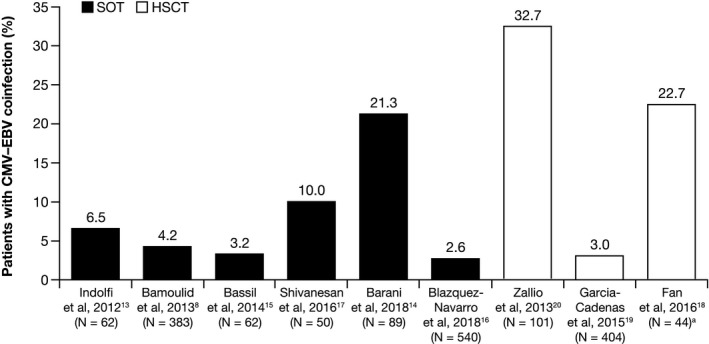
Post‐transplantation CMV‐EBV coinfection rates in SOT and HSCT recipients ^a^Cases also included in the separate CMV and EBV cohorts. CMV, cytomegalovirus; EBV, Epstein‐Barr virus; HSCT, hematopoietic stem cell transplant; SOT, solid organ transplant

The published rates of CMV‐EBV coinfection during the post‐transplantation period varied (Figure 2).

#### 3.2.1 SOT studies

In the study of pediatric liver transplant recipients, 6.5% (4/62) of patients had CMV‐EBV coinfections detected within the first 21 days after transplantation.^13^

Three of the five studies in renal transplantation detected low (≤5% of patients) CMV‐EBV coinfection rates.^8^, ^15^, ^16^ Of these, two studies monitored patients for 12 months post transplantation and found coinfection rates of 4.2% and 2.6%, respectively.^8^, ^16^ In the third study, 3.2% of patients followed for 36 months had CMV‐EBV coinfection^15^; however, the two cases reported were identified within 12 months post transplantation (at 8 and 10 months, respectively).^15^ Additionally, triple infection with BK polyomavirus (CMV‐EBV‐BK) was reported at a rate of 2.4%.^16^

The remaining two renal transplantation studies reported higher CMV‐EBV coinfection rates of 10.0%^17^ and 21.3%.^14^ In the latter study, infections were categorized as “immediate” (0‐3 months), “late” (>3‐12 months), or “very late” (>12 months) based on the time of assessment relative to transplantation.^14^ The rates of immediate, late, and very late coinfections were 24% (5/21), 17.6% (3/17), and 21.6% (11/51), respectively. That study, however, specifically included patients with symptoms suggestive of end‐organ disease, graft dysfunction, or renal dysfunction (without symptoms), who may have harbored a high risk for infection.^14^ In the study reporting a 10% CMV‐EBV coinfection rate, patients were monitored for viremia for 6 months; no additional cases of EBV DNAemia were reported after the 6‐month follow‐up period.^17^

#### 3.2.2 HSCT studies

The three HSCT studies reported CMV‐EBV coinfection rates of 3.0% (12/404),^19^ 22.7% (10/44),^18^ and 32.7% (33/101).^20^ In the study reporting the lowest rate (3.0%),^19^ the total study sample included patients who were not considered at high risk for EBV‐related complications, high‐risk patients, and those who were not at high risk but had developed moderate‐to‐severe steroid‐refractory graft‐vs‐host disease (GVHD). Although the number of patients with CMV‐EBV coinfection was reported for the cohort with EBV‐PTLD, it is unclear whether this represented all cases of coinfection (ie, CMV‐EBV coinfection without PTLD) and may have contributed to the lower reported rate of coinfection.^19^ In the study citing the highest rate of CMV‐EBV coinfection (32.7%; 33/101), all cases of coinfection had reactivation of both EBV and CMV (defined by viral load). This rate appeared consistent with the rates of reactivation of CMV (49%) and EBV (34%) monoinfection observed in the overall cohort.^20^

### 3.3 Clinical outcomes for transplant recipients with CMV‐EBV coinfection

Table 4 presents the rates and clinical outcomes reported for the most commonly cited outcomes of interest, graft dysfunction (rejection) or loss, and PTLD, for patients with CMV‐EBV coinfection.

**Table 4:** Studies reporting on graft rejection/loss or PTLD as post‐transplantation outcomes for CMV‐EBV coinfection

Study	Without coinfection	With CMV‐EBV
Graft rejection/loss, n/n (%)		
Indolfi G, et al 2012^13^	All other patients without CMV‐EBV coinfection: 18/58 (31.0)	2/4 (50.0)
Shivanesan P, et al 2016^17^	All other patients without CMV‐EBV coinfection: 4/45 (8.9)	1/5 (20.0)
Barani R, et al 2018^14^	All other patients without CMV‐EBV coinfection: 16/70 (23)	9/19 (47.4)
PTLD, n/n (%)		
Indolfi G, et al, 2012^13^	Patients with EBV monoinfection: 1/17 (5.9)	0/4 (0.0)
Bamoulid J, et al 2013^8^		0/16 (0.0)
Zallio F, et al 2013^20^	Whole cohort (N = 101): No cases reported	0/33 (0.0)
Bassil N, et al 2014^15^	Monoinfection cohorts: No cases reported	2/2 (100.0)
Garcia‐Cadenas I, et al 2015^19^	Patients with EBV monoinfection: 1/1 (100.0)	12/12 (100.0)
Shivanesan P, et al 2016^17^	Monoinfection cohorts: No cases reported	0/5 (0.0)
Blazquez‐Navarro A, et al 2018^16^	Whole cohort: 2/540 (0.4)	0/14 (0.0)

Abbreviations: CMV, cytomegalovirus; EBV, Epstein‐Barr virus; PTLD, post‐transplant lymphoproliferative disorders.

#### 3.3.1 Graft dysfunction or loss

Three studies specifically reported on the frequency of graft dysfunction, rejection, or loss among CMV‐EBV‐coinfected patients.^13^, ^14^, ^17^ In the study reporting the largest cohort with coinfection (n = 19 renal transplant recipients), graft dysfunction was documented in nearly half (47.4% [9/19]) of these cases.^14^ Overall, patients with coinfection comprised over one‐third (36.0% [9/25]) of the total number of cases with graft dysfunction in the study. Most patients (88.9% [8/9]) with CMV‐EBV coinfection and graft dysfunction had EBV DNAemia > 1000 copies/mL. Two patients with graft dysfunction had EBV DNAemia only (>1000 copies/mL).

In the second study, graft rejection occurred in 20.0% (1/5) of renal transplant recipients with CMV‐EBV coinfection and in 8.9% (4/45) of recipients without coinfection.^17^ The single patient with graft loss and coinfection had received pulse methylprednisolone and anti‐thymocyte globulin (ATG) for antibody‐mediated graft rejection, following which, they developed CMV infection. In that study, no case of graft loss occurred during the 6‐month post‐transplantation follow‐up period.^17^

In the third study, the incidence of acute graft rejection during the 21‐day post‐transplantation period did not differ between pediatric liver transplant recipients with CMV‐EBV coinfection (50% [2/4]) and those without coinfection (31% [18/58]).^13^ The two patients with coinfection and graft rejection comprised 10% (2/20) of the total number of cases with acute graft rejection. Both cases had received a second transplantation, following which, EBV and CMV viremia were detected in the first case at 7 days and 8 days, respectively, and in the second case at 2 months and 1 month, respectively. Both patients died from complications related to their underlying disease. Overall, no correlation was observed in the study between graft rejection and sex, age at transplantation, pre‐transplant EBV IgG and CMV IgG, and EBV and CMV viremia post transplantation.^13^

#### 3.3.2 PTLD

Of the seven studies that reported on PTLD, one liver transplantation study reported a single case of PTLD in the subgroup with EBV monoinfection only.^13^ A study in renal transplantation reported a severe case of PTLD in a patient with EBV monoinfection and a case of mild PTLD in a patient without EBV infection.^16^ Two studies in renal transplantation^8^, ^17^ and a single HSCT study^20^ reported no cases of PTLD during the study period (Table 4).

Two studies described cases of PTLD in renal and HSCT recipients with CMV‐EBV coinfection, respectively.^15^, ^19^ In the randomized controlled trial for belatacept, both renal transplant recipients (2/2) with CMV‐EBV viremia had been treated with belatacept and diagnosed with PTLD.^15^ PTLD in both cases was EBV‐related and involved the central nervous system (CNS). One patient was seropositive for both EBV and CMV at the time of transplantation and had reactivation of EBV and CMV at months 3 and 10, respectively. The patient was diagnosed with B‐cell CNS lymphoma by month 13 and died soon after. The second patient, who was EBV seropositive and CMV seronegative pre‐transplantation, had a CMV‐seropositive donor. That patient experienced a flare‐up of CMV disease at month 3 post transplantation and EBV reactivation at month 6. B‐cell CNS lymphoma was diagnosed by month 16. At the time of publication, the patient was alive and had a functioning graft.^15^ In the second study, patients with CMV‐EBV coinfection comprised 92.3% (12/13) of all cases with PTLD.^19^ Considering the total subgroup (n = 13), PTLD was diagnosed at a median (range) of 70 (31‐272) days post transplantation; only one case occurred after day 180. Eight cases had biopsy‐proven diffuse large B‐cell lymphoma, and five had probable disease. None had received rituximab prior to HSCT or sirolimus‐based GVHD prophylaxis.

### 3.4 Risk factors for CMV‐EBV coinfection

Table 5 presents a summary of risk factors for CMV and/or EBV infection and infection‐related clinical outcomes as reported in the studies.

**Table 5:** Summary of risk factors for CMV and/or EBV infection, and infection‐related clinical outcomes reported in the studies

	CMV pre‐infection/ reactivation	EBV pre‐infection/ reactivation	Positive CMV serostatus donor	Positive CMV serostatus donor/ recipient	Conditioning regimen	GVHD prophylaxis^a^	Second transplant
CMV‐EBV coinfection	– (pre‐infection)^18^	– (pre‐infection)^18^	↑ (OR, 3.97, *P* = .0127)^16^	↑^17^			
CMV infection/ disease		↑ (reactivation, *P = .187)* ^8^				↑ (OR: 13.6, *P = .040)* ^18^	
EBV infection					↑ (OR: 7.690, *P* = .034)^18^	↑ (OR: 23.7, *P = .013)* ^18^	↑ (HR: 2.6, *P = .04)* ^19^
EBV‐PTLD	– (reactivation)^19^/↑ (reactivation)^b^ ^,^ ^20^						↑ (HR: 6.4, *P = .02)*

Abbreviations: CMV, cytomegalovirus; EBV, Epstein‐Barr virus; GVHD, graft‐vs‐host disease; HR, hazard ratio; OR, odds ratio; PTLD, post‐transplant lymphoproliferative disease.

^a^ Includes prednisone.

^b^ CMV reactivation was a predictor of EBV reactivation and PTLD.

Three studies assessed the association between CMV reactivation and EBV in HSCT recipients. The study by Fan et al reported that pre‐infection with CMV or EBV did not significantly affect the rate of coinfection with EBV or CMV, respectively.^18^ However, conditioning regimens including ATG (*P* = .034) and GVHD regimens including prednisone (*P* = .013) were significantly associated with an increased risk of EBV infection. GVHD prophylaxis regimens that included prednisone were also strongly associated with an increased risk of CMV infection (*P* = .040). The second study concluded that CMV reactivation was not a significant predictor of EBV‐PTLD among patients at high risk for EBV‐PTLD; however, a second transplant more than doubled the risk of both EBV reactivation (hazard ratio 2.6 [95% confidence interval: 1.1‐6.4]; *P* = .04) and PTLD (hazard ratio 6.4 [95% confidence interval: 1.3‐31.9]; *P* = .02).^19^ By contrast, a multivariate analysis found that CMV reactivation was the most important predictor of EBV reactivation and the risk of PTLD. Other variables, including sex, conditioning, rituximab, donor serostatus, GVHD, and ATG, exhibited no significant predictive effects.^20^

Two studies reported a relationship between donor/recipient serostatus and the propensity for CMV‐EBV coinfection in renal transplant recipients. In the first study, all five CMV‐EBV coinfections occurred in donor‐positive/recipient‐positive cases.^17^ The second study identified donor CMV seropositivity as the only clinical variable significantly associated with combined CMV‐EBV reactivation (*P* = .0127).^16^ CMV‐EBV coinfection was also significantly associated with CMV and EBV viral loads greater than detectable levels (250 copies/mL) (*P* = .0237) and with elevated CMV and EBV DNAemia (*P* = .0416).^16^

Another study found that CMV disease occurred slightly more frequently in renal transplant recipients with EBV reactivation.^8^

### 3.5 Timing of coinfection post transplantation

The time interval between transplantation and the detection of CMV‐EBV coinfection was not the focus of the studies included in the SLR. However, a study of renal transplant recipients found that the subgroup presenting during the “immediate” post‐transplantation period featured a higher proportion of patients with CMV‐EBV coinfection (24%) compared with the subgroups presenting during later periods (<22% of patients).^14^ A study of HSCT recipients with coinfection reported that the median time between CMV and EBV infection was 26 days.^20^

In the two studies reporting the order of detection of CMV and EBV in patients with coinfection, detection of EBV preceded that of CMV in approximately 50% of patients in each study (Table 6).^16^, ^18^ One study reported only one case of concomitant detection of CMV and EBV.^18^

**Table 6:** Data reported on the time to diagnosis of post‐transplant infection for CMV and EBV

Study	Time to CMV	Time to EBV
SOT studies		
Indolfi G, et al 2012^13^	Mean (SD) time post transplant to CMV viremia: Cases with primary infection, 13.5 (10.6) d Cases with reinfection/reactivation, 13.3 (4.1) d	Mean (SD) time post transplant to EBV viremia: Cases with primary infection, 8.1 (3.5) d Cases with reinfection/reactivation, 7.7 (4.0) d
Bamoulid J, et al 2013^8^	NR	Median time to EBV DNAemia, 31 d post transplant
Bassil N, et al 2014^15^	Median (range) time from transplant to first positive CMV DNAemia: Belatacept group (n = 42), 6 (1‐22) mo CSA group (n = 20), 6 (1‐24) mo	Median (range) time from transplant to first positive EBV DNAemia: Belatacept group (n = 42), 3 (0.5‐35) mo CSA group (n = 20), 2 (1‐36) mo
Shivanesan P, et al 2016^17^	Subclinical infection in 16/17 (94%) cases occurred within 3 mo post transplant	Subclinical infection in 10/10 (100.0%) cases occurred within 3 mo post transplant
Barani R, et al 2018^14^	NR	By post‐transplant period^a^: ‐ Immediate: 6/21 (28.6%) ‐ Late: 7/17 (41.1%) ‐ Very late: 22/51 (43.1%)
Blazquez‐Navarro A, et al 2018^16^	Median time to first detectable viremia (IQR): 66 (54‐185) d (n = 92) CMV was detected before EBV in 29.6% of patients (n = 14 patients with CMV‐EBV)	Median time to first detectable viremia (IQR): 27 (7‐80) d (n = 109) EBV was detected before CMV in 51.9% of patients (n = 14 patients with CMV‐EBV)
HSCT studies		
Zallio F, et al 2013^20^	NR	Median (range) time to reactivation post transplant: 62 (4‐441) d
Garcia‐Cadenas I, et al 2015^19^	NR	Cohort at high risk for EBV‐related complications Median (range) time to EBV DNAemia post transplant: 42 (25‐281) d (n = 22) Median (range) time to EBV‐PTLD diagnosis: 70 (31‐272) d
Fan J, et al 2016^18^	Median (range) time to CMV DNAemia post transplant: 32 (11‐76) d post transplant (n = 20) CMV was detected before EBV in 4/10 (40%) CMV‐EBV patients	Median (range) time to EBV DNAemia post transplant: 45 (14‐88) d (n = 22) EBV was detected before CMV in 5/10 (50%) CMV‐EBV patients

Abbreviations: CMV, cytomegalovirus; CSA, cyclosporine A; d, day; EBV, Epstein‐Barr virus; HSCT, hematopoietic stem cell transplantation; IQR, interquartile range; mo, month; NR, not reported; PTLD, post‐transplant lymphoproliferative disease; SD, standard deviation; SOT, solid organ transplant.

^a^ Immediate, 0‐3 mo post transplant; late, >3 mo–12 mo post transplant; very late, >12 mo post transplant.

Eight studies disclosed the time of detection for CMV or EBV monoinfections post transplantation. Regardless of transplant type, the majority of infections (defined either by DNAemia or clinical symptoms) were detected within the first 3 months post transplantation (Table 6).^8^, ^13^, ^14^, ^16^, ^17^, ^18^, ^19^, ^20^ Only one study (renal transplantation) found that EBV DNAemia was detected in a higher proportion of patients assessed >1 year post transplantation than in those evaluated during earlier time periods.^14^ However, patients were not followed longitudinally, and their serostatus prior to presentation was unknown.

Among the four studies reporting the timing of both CMV and EBV monoinfections, the study in liver transplantation reported the shortest average times to initial detection of EBV and CMV viremia post transplantation with similar mean times for patients with primary infection and those with reinfection/reactivation (<2 weeks to CMV viremia; ~1 week to EBV viremia).^13^ A second study reported evident subclinical infections within the first 3 months after renal transplantation for all recipients with EBV infection (100% [10/10]) and for most of those with CMV infection (94% [16/17]).^17^ In a study in HSCT recipients, the median time from HSCT to the detection of viremia was 45 days and 32 days for patients with EBV and CMV infection, respectively.^18^ In another HSCT study, the median time to EBV viremia post transplantation was 42 days for patients at high risk for EBV‐PTLD; furthermore, 12 of 13 cases of EBV‐PTLD had reactivation of CMV.^19^

## 4 DISCUSSION

The clinical burden of CMV‐EBV coinfections in the transplant population is not well documented in the current literature. Over the 8‐year publication period studied, only nine studies on this topic were identified, and no identified study a priori set out to evaluate the rates of CMV‐EBV coinfection or to examine how CMV‐EBV coinfection status affects clinical outcomes. Limited data from the publications identified revealed highly variable CMV‐EBV coinfection rates (2.6%‐33.0%). It was apparent, however, that the impact that CMV and EBV infections had on clinical outcomes, the viability of the allograft, and the development of PTLD represented a common primary concern for healthcare providers and in terms of patient outcomes. Despite these concerns, guidelines for the management of CMV and EBV monoinfection in transplant recipients do not include recommendations for the treatment of coinfections.^21^, ^22^, ^23^, ^24^

Graft dysfunction/loss is a major concern in transplant patients. Among the three studies reporting graft dysfunction/loss in cases with coinfection, patients with CMV‐EBV coinfection comprised 10%‐36% of the total number of patients with graft dysfunction/loss. Both CMV and EBV infection have been implicated in the induction of graft rejection.^25^, ^26^ For example, in the case of early rejection episodes (<2 months post transplantation), EBV induced a cytotoxic T‐lymphocyte response through a phenomenon of allo‐cross‐reactivity.^25^ EBV‐PTLD presenting as allograft dysfunction has also been described.^27^ There is also a bidirectional relationship between CMV and graft rejection.^2^ Allograft rejection can trigger CMV reactivation post transplantation,^28^, ^29^ and CMV upregulates antigens resulting in alloreactivity.^2^ However, in the absence of a formal risk assessment, whether CMV‐EBV coinfection increases the risk of graft dysfunction/loss over either CMV or EBV monoinfection or no infection is unclear, and this SLR, therefore, highlights an unmet need for further research.

While results within the identified studies reporting on the rate of PTLD showed a trend toward greater risk among CMV‐EBV‐coinfected patients, those studies had a relatively short follow‐up time. Among the nine studies reviewed, two reported a total of 14 cases of PTLD with CMV‐EBV coinfection. The latency between transplantation and PTLD diagnosis was 13 months and 16 months, respectively, for the two cases in one study,^15^ while the median latency time for the remaining 12 cases in the second study was around 2 months (median [range] 70 [31‐272] days).^19^ Based on the current understanding, it is likely that with longer follow‐up and targeted investigation of patients with EBV‐PTLD, the frequency of CMV‐EBV coinfection would be higher. Older studies (outside of our search time period) that examined selected cohorts with PTLD also identified cases of CMV‐EBV coinfection.^30^, ^31^ A study of 20 heart transplant recipients found that two of the three patients who developed EBV‐positive PTLD had a history of CMV infection (mean time [range] to detection of PTLD: 56 [27‐84] months).^30^ In an earlier study, 7 out of 13 liver transplant recipients who experienced EBV seroconversion and developed PTLD had CMV disease prior to the diagnosis of PTLD.^31^ In that study, patients were diagnosed with PTLD up to 25.5 months (776 days; median 126 days) following transplantation.^31^

The studies identified in this SLR varied in terms of study design, study samples, clinical practices, post‐transplantation monitoring protocols, and follow‐up duration. There was also considerable variation in the pre‐ and post‐transplantation variables reported, including the duration and frequency of monitoring for viremia. Owing to these differences, a meta‐analysis of the data could not be performed. Other limitations include the following: the majority of the studies in the SLR were conducted at single centers; and while most studies were prospective, very few aimed to establish the rate of infections or examine the outcomes of coinfected patients. Furthermore, in the studies, assessment of the differences between groups involved limited hypothesis testing, and the analyses conducted were not adjusted for covariates, potentially affecting outcomes. These factors may have contributed to the variances reported across the studies, notably regarding differences in coinfection rates.

A key finding of this SLR was that CMV and EBV infections seem to have an impact on the viability of the allograft that differs from monoinfection, which together with the development of PTLD represent a primary concern for the management of transplant recipients. As knowledge of the interplay of HHVs in the transplant setting continues to evolve, additional research specifically evaluating the rate of coinfection and outcomes of CMV‐EBV coinfection is needed. A better understanding of differing outcomes based on coinfection status could provide optimal methods for applying antiviral therapies and strategies aimed at CMV‐EBV or other HHV combinations.

## CONFLICT OF INTEREST

CA‐S and IH are employees of, and hold stock in, Shire International GmbH, a Takeda company. ERB is an employee of CTI Clinical Trial and Consulting Services, which was funded by Shire, a Takeda company, to conduct this study.

## AUTHOR CONTRIBUTIONS

All authors contributed to the conception or design of the work and interpretation of the data. All authors contributed to drafting of the work and revising it critically for intellectual content. The manuscript was drafted, critically revised, and finally approved by all of the named authors.

## Supporting information

Supplementary Material

## Data Availability

All data reported in this systematic literature review were obtained from the published literature.
