# Supplementary material for: Coinfection rates and clinical outcome data for cytomegalovirus and Epstein‐Barr virus in post‐transplant patients: A systematic review of the literature
Source: Transpl Infect Dis. 2020 Jul 27;22(6):e13396. doi: 10.1111/tid.13396 (PMC7816247; doi:10.1111/tid.13396)
Supplement: Supplementary file 1 — Supplementary Material [file TID-22-e13396-s001.docx]

**SUPPLEMENTARY INFORMATION**

**SUPPLEMENTARY MATERIALS AND METHODS**

Pre-specified study-level data extracted during reference screening included:

- Study design
- Characteristics of the cohort and transplantation
- Use and types of immunosuppressive therapies
- Antiviral prophylaxis and pre-emptive therapies
- CMV and EBV (CMV–EBV) coinfection rates
- The time of detection of coinfection following the transplant procedure
- Viral loads
- Clinical outcomes associated with CMV–EBV coinfection

**SUPPLEMENTARY TABLE 1** Details of patient and treatment characteristics pre-transplantation and post-transplantation outcomes

| **Study**  **(transplant type)** | **Pre-transplant** | **Post-transplant** | | | |
| --- | --- | --- | --- | --- | --- |
|  |  | **Antiviral measures and monitoring** | **Infection rates, time to diagnosis, clinical outcomes** | | |
|  |  |  | **CMV** | **EBV** | **CMV–EBV** |
| Indolfi G, et al. 2012  (Liver)  N = 62 | **CMV/EBV serostatus:**  EBV status: (N = 62)  IgG (−): 24 (39%)  IgG (+): 19 (30%)  Indeterminate: 19 (30%)  CMV status: (N = 62)  IgG (−): 28 (45%)  IgG (+): 14 (23%)  Indeterminate: 20 (32%)  **Immunosuppression:**  No induction  Maintenance: tacrolimus | **Antiviral prophylaxis:**  Antibacterial and antifungal prophylaxis × ≥5 d post-T  Ganciclovir for CMV D+/R−  **Monitoring:**  Total follow-up: 21 d  qPCR once per week on same day each week  Median (IQR) follow-up: 28.2 (6.6) months  **Viremia/infection definition:** NR | 11 (17.7%) within 21 d post-T  Primary: n = 2  Reactivations/reinfections: n = 4  Indeterminate: n = 5  Mean (SD) time post-T to CMV viremia was similar between cases with primary infection (13.5 [10.6] d) vs. reinfection/reactivation (13.3 [4.1] d)  Viral load (copies/mL)^a^:  Primary infection (n = 2): 456 and 690  Reactivation/reinfection  (n = 4): mean, 6233  **Clinical outcomes:**  Acute rejection: 3 (27.3%)  PTLD: 0  Death: 0 | 17 (27.4%) within 21 d post-T  Primary: n = 3  Reactivations/ reinfections: n = 13  Indeterminate: n = 1  Similar time (mean [SD]) post-T to EBV viremia between patients with primary infection (8.1 [3.5] d) vs. reinfection/  reactivation (7.7 [4.0] d)  Mean viral load (copies/mL)^a^:  Primary infection (n = 3): 560,886  Reactivation/  reinfection (n = 13): 140,468  **Clinical outcomes:**  Acute rejection: 7 (41.2%)  PTLD: 1 (5.9%)  Death: 2 (11.8%) (acute rejection, n = 1; PTLD, n = 1) | 4 (6.5%) in 21 d post-T  No significant differences in age, AST, γ-GT, ALP, and bilirubin levels between CMV–EBV patients vs. other patients  **Clinical outcomes:**  Acute rejection: 2 (50%)  PTLD: 0  Death (acute rejection): 2 (50%)  (Patients with no coinfection [N = 58]:  Acute rejection: 18 [31.0%];  Deaths [see EBV]: 2 [3.4%]) |
| Bamoulid J, et al. 2013  (Kidney)  N = 383 | **CMV/EBV serostatus:**  357 patients were EBV seropositive pre-T  EBV serostatus: n (%)  D+/R+: 311 (87.1%)  D−/R+: 46  D+/R−: 18 (4.7%)  D−/R−: 8  **Immunosuppression:**  Induction: ATG or anti-CD25  Maintenance: ATG/anti-CD25+ MMF, tacrolimus, steroids | **Antiviral prophylaxis:**  For CMV R+ or D+/R− VGCV, SMZ-TMP × 3 months post-T  For EBV D+/R−: IVIG × 6 months post-T  **Monitoring:**  Total follow-up: 12 months  PCR q2w × 3 months → q4w × 9 months  **CMV disease definition:** Viral replication and clinical symptoms | 31 (8.1%)  Slightly more frequent CMV disease in patients with EBV reactivation (10.3% vs. 6.6%;  *P* = 0.187)  **Clinical outcomes:**  Graft rejection/loss: NR  PTLD: 0 | 155 (40.1%)  Median (range) time to EBV DNAemia:  31 (14–329) d post-T  % with first positive viremia by time of detection:  <1 month: 49%  1–3 month: 35%  3–6 month: 5%  >6 month: 9%  70 (18.3%) patients had persistent viremia for >6 months. Viral load was highly predictive of persistent viremia after first y post-T (*P* < 0.0001)  No difference between patients with and without EBV viremia based on age, sex, underlying renal disease, type of transplant, or CMV serostatus  **Clinical outcomes:**  ≥1 episode of severe acute bacterial infection: 21.3%  Opportunistic infections: 11.6%  Graft loss: 27 (20.3%)  PTLD: 0 | 16 (4.2%)  **Clinical outcomes:**  Graft rejection/loss: NR  PTLD: 0 |
| Barani R, et al. 2018  (Kidney)  N = 89 | **CMV/EBV serostatus:** NR  **Immunosuppression:** NR | **Antiviral prophylaxis:**  NR  **Monitoring:**  qPCR at study enrolment  **Viremia/infection definition:** DNAemia ± clinical symptoms at time of test (“onset”) post-T, categorized by post-T period:  Immediate: 0–3 months  Late: >3–12 months  Very late: >1 y  Total n (%) by post-T period:  Immediate: 21 (23.6%)  Late: 17 (19.1%)  Very late: 51 (57.3%) | 19 (21.3%); based on number of coinfection patients    CMV monoinfection: NR  By post-T period: NR for CMV monoinfection  **Clinical outcomes:**  Graft dysfunction: 2 (10.5%)  PTLD: NR | 35 (39.3%)  By post-T period:  Immediate: 6/21 (28.6%)  Late: 7/17 (41.1%)  Very late: 22/51 (43.1%)  EBV viral load, range: 324–32,436 copies/mL  Mean EBV viral load (SD) (copies/mL) by time of onset:  Immediate (n = 6): 6081 (7863)  Late (n = 7): 3118 (1359)  Very late (n = 22): 8029 (9560)  **Clinical outcomes:**  Graft dysfunction: 2 (5.7%)  PTLD: NR | 19 (21.3%)  By post-T period::  Immediate: 5/21 (24%)  Late: 3/17 (17.6%)  Very late: 11/51 (21.6%)  EBV DNAemia (mean [SD]) was lower in CMV–EBV patients vs. those with EBV DNAemia only (4581 [6279] copies/mL vs. 9600 [10,046] copies/mL, respectively), but not statistically different  **Clinical outcomes:**  Graft dysfunction: 9 (47%)  PTLD: NR  (Patients with no coinfection [N = 70]:  Graft dysfunction: 16 [23%]) |
| Bassil N, et al. 2014  (Kidney)  N = 62 | **CMV/EBV serostatus:**  CMV (+/−), n:  Belatacept (Group A)  (n = 42):  D: 24/18; R: 25/17  CSA (Group B) (n = 20):  D: 13/7; R: 10/10  EBV (+/−), n:  Group A: (n = 42):  D: 39/3; R: 38/4  Group B: (n = 20):  D: 18/2; R: 16/4  **Immunosuppression:**  Induction: basiliximab + MMF + steroid tapered by 6 months post-T  Maintenance: betalacept or CSA  Discontinued by 36 months:  Group A: 14%  Group B: 50%, *P* = 0.005 | **Antiviral prophylaxis:**  SMZ-TMP × 6 months  post-T  For CMV R+ or D+/R−: VGCV × 3 months post-T  **Monitoring:**  Total follow-up by PCR: 36 months  Group A: Monthly  Group B: Monthly × 6 months → quarterly  **Viral infection definition:**  +DNAemia  **Viral disease definition:**  +DNAemia with clinical symptoms | Total: 29/62 (47%) in 36 months  Group A: 20/42 (47.6%)  Group B: 9/20 (45.0%)  Primary CMV infection in D+/R− patients (%):  Group A: 54  Group B: 45  CMV reactivation in seropositive patients (%):  Group A: 76  Group B: 65  **Clinical outcomes:**  PTLD: 0  JC viremia: 4 (13.8%) | Total: 42/62 (68%) in 36 months  Group A: 32/42 (76.2%)  Group B: 10/20 (50.0%)  (*P* = 0.048 vs. Group A)  Primary EBV infection in D+/R− patients, n:  Group A: 2 of 3  Group B: 2 of 4  EBV reactivation in seropositive patients (%):  Group A: 78  Group B: 50  **Clinical outcomes:**  PTLD: 0  JC viremia: 4 (9.5%) | Total: 2/62 (3.2%) in 36 months  **Clinical outcomes:**  PTLD: 2 (100%); both cases occurred in belatacept-treated EBV-seropositive patients  Death (PTLD): 1 (50%)  JC viremia: 1 (50%) for patient with mixed EBV, CMV, and BKV infection  (Total cohort:  Graft rejection: 26 [41.9%]  Graft loss: 2 [3.2%]) |
| Blazquez-Navarro A, et al. 2018  (Kidney)  N = 540 | **CMV/EBV serostatus:** NR  **Immunosuppression:**  Induction: basiliximab or rabbit ATG  Maintenance: tacrolimus + MMF ± steroids | **Antiviral prophylaxis:**  For CMV/EBV D+/R−: VGCV × ≥3 months  **Monitoring:**  qPCR was performed at eight visits over 1-y period  post-T  **Viremia/infection definition:**  By detectable viral load for ≥1 visit:  CMV+: >250 copies/mL  EBV+: >250 copies/mL  By elevated viral load (eCMV, eEBV) for ≥1 visit:  eCMV: >2000 copies/mL  eEBV: >2000 copies/mL | Detectable viremia: 92 (17.0%)  Elevated viremia: 39 (7.2%)  Median time to first detectable viremia (IQR):  66 (54–185) d  n (%) with no detectable viremia at 1-y post-T (clearing): 61 (95.3%)  **Clinical outcomes:**  PTLD: 0  Graft rejection/loss: NR | Detectable viremia: 109 (20.2%)  Elevated viremia: 37 (6.9%)  Median time to first detectable viremia (IQR):  27 (7–80) d  n (%) with no detectable viremia at 1-y post-T (clearing): 48 (85.7%)  **Clinical outcomes:**  PTLD: 1 (0.9%)  Graft rejection/loss: NR | Detectable viremia:  14 (2.6%) had CMV–EBV  13 (2.4%) had CMV–EBV–BK  Elevated viremia:  5 (0.9%) had CMV–EBV  1 (0.2%) had CMV–EBV–BK  EBV was detected before CMV in 51.9%; CMV was detected before EBV in 29.6%  CMV–EBV coinfection was significantly associated with CMV+ donor (OR: 3.97; 95% CI: 1.35–11.6;  *P* = 0.0127)  **Clinical outcomes:**  PTLD: 0  Graft rejection/loss: NR  (Total cohort:  Graft loss at 1 y: 22 [4.1%]  PTLD: 2 [0.4%]  Death at 1 y: 16 [3%]) |
| Shivanesan P, et al. 2016  (Kidney)  N = 50 | **CMV/EBV serostatus:**  CMV+, %: D: 96%;R: 98%  D+/R+: 48 (96%)  D+/R−: 1 (2%)  D−/R−: 1 (2%)  EBV: NR  **Immunosuppression:**  Tacrolimus or CSA + MMF/azathioprine; basiliximab/ATG induction (patients with unrelated donors)  23 (46%) patients received induction therapy | **Antiviral prophylaxis:**  None  **Monitoring:**  Total follow-up: 6 months  Frequency of qPCR:  CMV (n = 50): 1, 2, 3, and 6 months post-T  EBV (n = 36): 1, 3, and 6 months post-T  Additional qPCR for symptomatic cases  **Viremia/infection definition:** NR | 17 (34%)  Subclinical infection in 16/17 patients occurred 3 months post-T:  First month: 4 (23%)  Second month: 6 (35%)  Third month: 6 (35%)  Pre-T seropositivity in infected cases: n (% of infected cases):  D+/R+: 16 (94%)  D+/R−: 1 (6%)  Viral load (mean [range] copies/mL):  Asymptomatic CMV  (n = 3): 81,300 (41,000–4,914,000)  Symptomatic CMV (n = 11): 600,000 (70,000–89,400,000) (*P* = NS)  **Clinical outcomes:**  Graft rejection: 4 (23.5%)  PTLD: 0 | 10 (20%)  Subclinical infection in 10/10 patients occurred <3 months post-T:  First month: 6 (60%)  Third month: 4 (40%)  No additional EBV DNAemia patients after 6 months of follow-up  Mean viral load (copies/  mL)^b^:  Asymptomatic EBV: 2436  Symptomatic EBV: 4,476,000  **Clinical outcomes:**  Graft rejection: 1 (10%)  PTLD: 0 | 5 (10%)  5 (100%) cases with CMV–EBV were D+/R+  One (6%) patient with CMV and EBV coinfection had antibody-mediated rejection; treated with pulse methylprednisolone and ATG; subsequent CMV infection  **Clinical outcomes:**  Graft rejection: 1 (20%)  PTLD: 0 |
| Fan J, et al. 2016  (HSCT)  N = 44 | **CMV/EBV serostatus:**  CMV: all recipients were seropositive (IgG+, IgM-)  EBV: NR  **Immunosuppression:**  ±ATG with MMF + CSA with short-term methotrexate, ganciclovir × 7 d pre-T  T-cell depletion reported | **Antiviral prophylaxis:**  SMZ + ganciclovir  **Monitoring:**  Weekly qPCR in first month → 2×/w in second month and third month until Dec 2012  Follow-up time, range: 3 months to 1 y  **Viremia/infection definition:** NR | 20 (45.5%)  Median (range) time to CMV DNAemia: 32 (11–76) d post-T    **Clinical outcomes:**  NR separately | 22 (50.0%)  Median (range) time to EBV DNAemia: 45 (14–88) d post-T  **Clinical outcomes:**  NR separately | 10 (22.7%); number of cases appear to be included in the separate cohorts with CMV and EBV  Five cases positive for EBV first; four for CMV, and one for EBV and CMV simultaneously  Two (4.5%) patients coinfected with CMV–EBV–HHV-6; both were positive for CMV, then EBV, and then HHV-6  Logistic regression: Pre-infection with EBV did not affect CMV infection rate  **Clinical outcomes:**  NR separately  (Total cohort:  Acute GVHD, grade 0–1: 36 [81.8%]  Acute GVHD, grade 2–4: 8 [13.6%]  Death: 5 [11.4%]; all were unrelated to viral infection) |
| Garcia-Cadenas I, et al. 2015  (HSCT)  N = 404 | **CMV/EBV serostatus:**  CMV D−/R−, n (%):  Baseline cohort: 18/93 (19.4%)  SR-GVHD cohort: 3/40 (7.5%)  EBV: NR  **Immunosuppression:**  Myeloablative conditioning (CY+TBI/BU) or reduced intensity conditioning; ATG for cases with mismatched unrelated donors  Conditioning regimen for cord-blood transplants  T-cell depletion during conditioning in 93.5% (87/93) of baseline cohort and 2.5% (1/40) of SR-GVHD cases | **Antiviral prophylaxis:**  Antiviral, antifungal, SMZ-TMP or nebulized pentamidine  PET (rituximab) for EBV >1000 copies/mL × 2 consecutive occasions or EBV >2000 copies/mL × 1  **Monitoring:**  Weekly qPCR for EBV DNAemia starting at time of HSCT in high-risk cases, and after starting second-line immunosuppressive therapy in SR-GVHD cases (discontinued if no reactivation after first 6 months)  Median duration (range) of monitoring: 180 (15–557) d  Median duration (range) of follow-up: 604 (34–2204) d  **Definition of EBV reactivation**:  Any EBV viral load >1000 copies/mL | CMV monoinfection: NR  12 of 13 (92.3%) of patients with EBV-PTLD had CMV reactivation  **Clinical outcomes:** NR | Total sample: 22/404 (5.4%)  High-risk cohort (for EBV-related complications): 22/93 (23.7%)  Cumulative incidence:  6 months: 27.4% (95% CI: 22.2–32.6)  1 y: 29.4% (95% CI: 18.4–40.5)  Median (range) time from HSCT to EBV DNAemia: 42 (25–281) d. Most occurred within first 3 months post-T; 5 (22%) after day 100  Steroid-refractory GVHD cohort: 6/40 (15%)  Median (range) time from HSCT to EBV DNAemia: 82 (25–298) d  Cumulative incidence:  6 months: 14.8% (95% CI: 7.9–21.7)  12 months: 31.8% (95% CI: 19.7–43.9)  **Clinical outcomes:**  PTLD: 1 | 12/404 (3%); based on number of EBV-PTLD cases with CMV reactivation reported for total sample  Multivariate analysis (n = 93): CMV reactivation was not a significant predictor of EBV-PTLD  **Clinical outcomes:**  PTLD: 12 (100%; coinfection patients represent EBV-PTLD patients with CMV reactivation)  PTLD symptoms (n = 12):  B-symptoms: 12 (92%)  Extranodal involvement: 10 (77%)  Tonsillar involvement: 6 (46%)  Altered liver function tests: 8 (62%)  BK-polyomavirus hemorrhagic cystitis: 5 (38%)  Acute GVHD, grade 2–4 at PTLD diagnosis: 6 (46%) |
| Zallio F, et al.  2013  (HSCT)  N = 101 | CMV serostatus (n = 100), n (%):  D−/R−: 20 (20%)  D−/R+: 24 (24%)  D+/R+: 33 (33%)  D^+^/R−: 23 (23%)  Immunosuppression:  Myeloablative conditioning (n = 53) or reduced intensity conditioning (n = 48)  *In vivo* T-cell depletion: n = 46  GVHD prophylaxis with CSA + methotrexate or MMF | **Antiviral prophylaxis:**  Anti-CMV PET in cases with ≥1 positive cell/200,000 leukocytes confirmed × 2  **Monitoring:**  EBV: weekly qPCR 3 months post-T. If GVHD after day 100 → to end of immunosuppressive therapy  Positive qPCR: EBV DNAemia weekly × ≥2 months or until end of immunosuppressive therapy  CMV: weekly qPCR 2×/w → post-T day 100 and as clinically indicated  Median (range) follow-up: 13 (0–78) months  PET (rituximab) for patients at high risk for PTLD (n = 13)  **Viral load threshold definitions:**:  EBV reactivation: >500 copies/mL  High-risk for PTLD: >10,000 copies/mL | 50 (49.5%) had CMV reactivation  **Clinical outcomes:**  GVHD: NR  PTLD: 0 | 34 (34%) had EBV reactivation  Median (range) time from HSCT to reactivation: 62 (4−441) d  13 patients at high risk for PTLD received PET (rituximab) at a median (range) of 72 (43−526) days post-T  All patients cleared EBV DNAemia without recurrence  **Clinical outcomes:**  GVHD: 18 (52.9%)  PTLD: 0 | 33 (33%) had reactivation of both EBV and CMV  Median time between CMV and EBV reactivation: 26 d  Multivariate analysis: CMV reactivation was most important predictor of EBV reactivation and risk for PTLD  **Clinical outcomes:**  GVHD: NR  PTLD: 0    (Total cohort:  GVHD: 40 [39.6%]  PTLD: 0) |

^a^SD was not reported.

^b^Range was not reported.

γ-GT, gamma glutamyl transferase; ALP, alkaline phosphatase; AST, aspartate aminotransferase; ATG, anti-thymocyte globulin; BKV, BK virus; BU, busulfan; CD, cluster of differentiation; CI, confidence interval; CMV, cytomegalovirus; CSA, cyclosporine A; CY, cyclophosphamide; d, day; D, donor; EBV, Epstein–Barr virus; GVHD, graft-versus-host disease; HHV-6, human herpesvirus 6; HSCT, hematopoietic stem cell transplantation; Ig, immunoglobulin; IQR, interquartile range; IVIG, intravenous immunoglobulin; JC, John Cunningham; MMF, mycophenolate mofetil; NR, not reported; NS, not significant; OR, odds ratio; PCR, polymerase chain reaction; PET, pre-emptive therapy; post-T, post-transplant; pre-T, pre-transplant; PTLD, post-transplant lymphoproliferative disease; q2w, every 2 weeks; q4w, every 4 weeks; qPCR, quantitative polymerase chain reaction; qRT-PCR, quantitative real-time polymerase chain reaction; R, recipient; SD, standard deviation; SMZ-TMP, sulfamethoxazole-trimethoprim; SR-GVHD, steroid-refractory graft-versus-host disease; TBI, total body irradiation; VGCV, valganciclovir; w, week; y, year.
